# Leukocyte Rho kinase activity and serum cystatin C affect cardiovascular events in acute coronary syndrome

**DOI:** 10.1097/MD.0000000000020060

**Published:** 2020-07-10

**Authors:** Li Ma, Wenqin Dai, Yongbo Lin, Zhongyuan Zhang, Yunhong Pan, Hongyan Han, Haizhen Jia, Jun Peng, Jinhe Zhao, Liang Xu

**Affiliations:** a Department of Cardiology, Tianyou Hospital Affiliated to Wuhan University of Science and Technology; b Department of Cardiology, Wuchang Hospital of Wuhan; c Department of Cardiology, People's Hospital of Dongxihu; d Department of Pharmacy, Tianyou Hospital Affiliated to Wuhan University of Science and Technology; e Department of Intensive Care Unit, WuHan, China.

**Keywords:** acute coronary syndrome, cardiovascular events, cystatin C, Rho kinase activity

## Abstract

**Objective:**

This study was designed to investigate the effects of leukocyte Rho kinase activity and serum Cystatin C (Cys C) on cardiovascular events in patients with acute coronary syndrome (ACS).

**Methods:**

A total of 48 patients with ST-segment elevation myocardial infarction (STEMI), 23 patients with non-ST-segment elevation myocardial infarction (NSTEMI), 25 patients with unstable angina (UA) and 20 patients with no-acute coronary syndrome as control from January 2017 to June 2018 in Tianyou Hospital affiliated to Wuhan University of Science and Technology were selected in this study. Western blot was used to detect the leukocyte Rho kinase activity and Elisa kit was used to measure serum Cys C. Univariate and multivariate analysis were used to analyze the influencing factors of cardiovascular events in ACS patients.

**Results:**

The activity of leukocyte Rho kinase and serum Cys C were gradually reduced in the STEMI, NSTEMI and UA patients, but all significantly higher than that in No-ASC patients, and there was a positive correlation between leukocyte Rho kinase activity and serum Cys C in ACS patients (r = 0.516, *P* < .001). The activity of leukocyte Rho kinase was positively correlated with the levels of serum TNF-α (r = 0.634, *P* < .001), IL-6 (r = 0.578, *P* < .001), IL-8 (r = 0.582, *P* < .001) in ACS patients, and the level of Cys C was positively correlated with the levels of serum TNF-α (r = 0.634, *P* < .001), IL-6 (r = 0.578, *P* < .001), IL-8 (r = 0.582, *P* < .001) in ACS patients. Univariate and multivariate analysis showed that the leukocyte Rho kinase activity (HR = 2.994, 95%CI = 1.328–6.054, *P* < .0001) and the levels of serum Cys C (HR = 1.692, 95%CI = 1.028–2.124, *P* < .0001) were independent influencing factors of cardiovascular events in ACS patients.

**Conclusion:**

The leukocyte Rho kinase activity and serum Cystatin C are high in acute coronary syndrome patients, and are the independent influencing factors of cardiovascular events in ACS patients.

## 1 Introduction

Acute coronary syndrome (ACS) is a group of clinical syndromes caused by acute myocardial ischemia, which is an acute illness of coronary heart disease, including ST-segment elevation myocardial infarction (STEMI), non-ST-Segment elevation myocardial infarction (NSTEMI) and unstable angina (UA).^[1,2]^ Although the establishment of chest pain clinic and the improvement in ACS treatment has improved the survival rate of ACS patients in China, recurrent cardiovascular events are still the main cause of death in ACS patients.^[1,2]^ Therefore, the prevention of recurrent cardiovascular events in ACS patients has become more and more important to improve the he survival rate of ACS patients. Previous studies have found that pulse wave velocity, left atrial volume index, serum resistin, thyroid hormone, and CRP levels affect and predict cardiovascular events in ACS patients, and correcting these factors in advance can reduce the occurrence of cardiovascular events.^[3]^

Rho kinase is one of the downstream targets of the small GTP-binding protein RhoA and plays a key role in vascular smooth muscle contraction, endothelial function and many cell regulation.^[3]^ Multicenter evidence suggested Rho kinase activity in patients with angina pectoris, vasospasm angina, pulmonary hypertension, heart failure, microangiopathy, and cerebral infarction were increased, and Rho kinase might play a key role in the pathogenesis, progression and prognosis of several cardiovascular diseases.^[4]^ Cystatin C (Cys C) is the most important endogenous cysteine protease inhibitor outside the cell and has been shown to be a sensitive indicator of renal function,^[5]^ and has also been found to be closely related to the occurrence of cardiovascular disease in recent years.^[6]^ Previous study have indicated that serum cystatin C levels in patients with coronary heart disease could be used to determine the severity of coronary heart disease.^[6]^

In the present study, we compared leukocyte Rho kinase activity and serum Cystatin C in STEMI, NSTEMI, UA, and No-ACS patients, and analyzed their effects on recurrent cardiovascular events in patients with ACS. We found that the leukocyte Rho kinase activity and serum Cystatin C were high in acute coronary syndrome patients, and were independent influencing factors of cardiovascular events in ACS patients. All in all, our study indicated that leukocyte Rho kinase activity and serum Cystatin C affected cardiovascular events in acute coronary syndrome, and intimated leukocyte Rho kinase activity and serum Cystatin C were potential targets for reducing cardiovascular events in ACS patients.^[7]^ Although a large number of clinical studies have shown that Cys C is involved in atherosclerosis and thrombosis and is one of the independent risk factors for cardiovascular and cerebrovascular diseases,^[8,9]^ no studies have reported that Cys C is involved in the regulation of intimal proliferation or is related to leukocyte Rho kinase activity. To further investigate the rational relationship between Cystatin C and Rho kinase, we analyzed the association of the activity of leukocyte Rho kinase and serum Cys C with serum inflammatory factors in patients with ACS. We found that the activity of leukocyte Rho kinase and serum Cys C was positively correlated with serum inflammatory factors (TNF-α, IL-6, IL-8), suggesting that the activity of leukocyte Rho kinase and serum Cys C are associated with inflammation in patients with ACS.

## 2 Patients and methods

### 2.1 Sample size calculation and ethics statement

The present study was performed with the approval of the Ethics Committee of the Tianyou Hospital affiliated to Wuhan University of Science and Technology. All aspects of the study complied with the Declaration of Helsinki. In addition, all participants signed the informed consent.

As a single center study, the primary factor for comparison is leukocyte Rho kinase activity or serum Cystatin C at first event, that is 2 endpoint for a research subject. Therefore, 10 times the endpiont is the smallest sample size (n = 20). At second event, the power for the primary endpoint cardiovascular event is calculated based on a two sided *t* test with a significance level of 5%. And the main research factors that affect the primary endpoint in this study is gender, age, complications, treatment, blood lipids, blood glucose, leukocyte Rho kinase activity and serum Cystatin C. Therefore, 10 times the endpiont is the smallest sample size (n = 70).

### 2.2 Patients

A total of 116 patients who underwent coronary angiography in the Tianyou Hospital affiliated to Wuhan University of Science and Technology from January 2018 to December 2018 due to chest pain or chest discomfort, and there were 96 ACS patients and 20 healthy patients from checkup are chosen as control group in this study (Table 1). A total of 96 patients with ACS were further divided into STEMI group (n = 48), NSTEMI group (n = 23) and UA group (n = 25). STEMI patients was diagnosed according to the ACC/AHA 2009 Guidelines for the Treatment of Acute Myocardial Infarction.^[10]^ NSTEMI and UA patients were diagnosed with reference to the ACC/AHA 2007 UA/NSTEMI guidelines,^[11]^ and No-ACS patients were determined based on coronary angiographic results. In addition, patients with thyroid disease, stroke, rheumatic heart disease, acute and chronic infectious diseases (such as lung infections), connective tissue diseases, immune diseases, malignant tumors, blood system diseases, other heart diseases such as dilated cardiomyopathy, viral Myocarditis, etc. are excluded and patients with Renal function creatinine greater than 225 umol/L or liver function transaminase greater than 120U / L are also excluded.

**Table 1:**
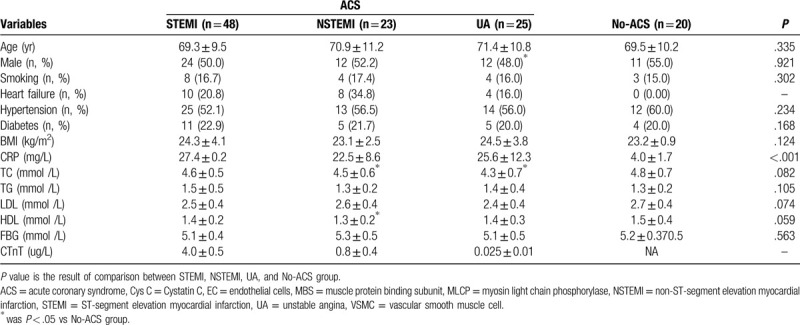
Patient characteristics of the cross-sectional study.

### 2.3 Blood samples and plasma index

A total of 10 to 15 mL of peripheral blood was drawn and serum was collected by centrifugation (1000 g, 10 minutes, room temperature). Blood glucose meter (EA-12, Sinocare, China) is used to measure fasting blood glucose (FBG) and automated biochemical analyzer (AU5800, BECKMAN, USA) for measuring serum total cholesterol (TC), triglycerides (TG), high density lipoprotein (HDL) and low density lipoprotein (LDL).

Bovine C Reactive Protein (CRP) ELISA Kit was used to detect the level of serum CRP (20182402374, Wuhan Easydiagnosis Biomedicine Co., Ltd, China), human (cTnT) ELISA assay kit (YM-SX1142, Shanghai Yuanmu Biotechnology Co., Ltd., China) for the level of serum cTnT, Human IL-8 Assay ELISA Kit (H-EL-IL-8, ZYscience, USA) for human IL-8; Human IL-6 Assay ELISA Kit (H-EL-IL-6, ZYscience, USA) for human IL-6; Human TNF-α Assay ELISA Kit (50R-E.1693H, BIOVALUE, AUS) for human TNF-α. And Human Cystatin C ELISA Kit (RAB0105, SIGMA, USA) for serum Cystatin C.

### 2.4 Separation of leukocyte and western blot

Human Peripheral Blood Leukocyte Separation Kit (P8670, Solarbio) is used to isolate leukocyte as describe previously and instruction manual.^[12]^ RIPA Lysis Buffer (P0013K, Beyotime, Shanghai, China) was used to lyse leukocytes, and BCA Protein Assay Kit (P0010S, Beyotime, Shanghai, China) was used to measure lysate protein concentration. Total of 50 ug protein in tissue or cell lysates were separated by 10% SDS-page and then transferred to PVDF membrane, and blocked with 5% skim milk powder for 1 hour at room temperature. Primary antibody: Anti-Myosin Phosphatase antibody (1:500, ab59235, ABCAM, UK), Anti-Myosin Phosphatase (phospho T853) antibody (1:500, ab59203, ABCAM, UK) and Anti-beta Actin antibody (1:3000, ab8227 ABCAM, UK). Second antibody: goat anti-rabbit (1:1000, ab150077, ABCAM, UK), or goat anti-rat (1:1000, ab150117, ABCAM, UK). Primary antibody was incubated overnight at 4°C and second antibody was incubated for 1 hour at roomtemperature. BeyoECL Plus kit (P0018S, Beyotime, ShangHai, China) was used to chromogenic and densitometry protein bands with Beckman Coulter Immunoassay System (UniCel DxI 800, Beckman, CA).

### 2.5 Statistical analyses

Statistical Product and Service Solutions 20.0 (IBM, USA) was used to analysis the data in the present study. Data between the 2 groups were compared by student's *t* test or chi-square test, and multiple groups was compared with one-way ANOVA that duncan test as post hoc test. Pearson method was used to analyze the correlation between 2 group data, and logistic regression models was constructed to determine the hazard ratio (HR) and 95% confidence interval (CI) for putative risk factors associated with cardiovascular events in ACS patients. *P* < .05 means significant difference.

## 3 Results

### 3.1 Leukocyte Rho kinase activity in patients with or without ACS

Total of 48 STEMI patients, 23 NSTEMI patients, 25 UA patients and 20 No-ACS patients were chosen in the present, and their clinical data was showed in Table 1 ——there was no significant difference in the comparison of other clinical data between ACS patients and No-ACS patients except for serum CRP levels.

**Figure 1:**
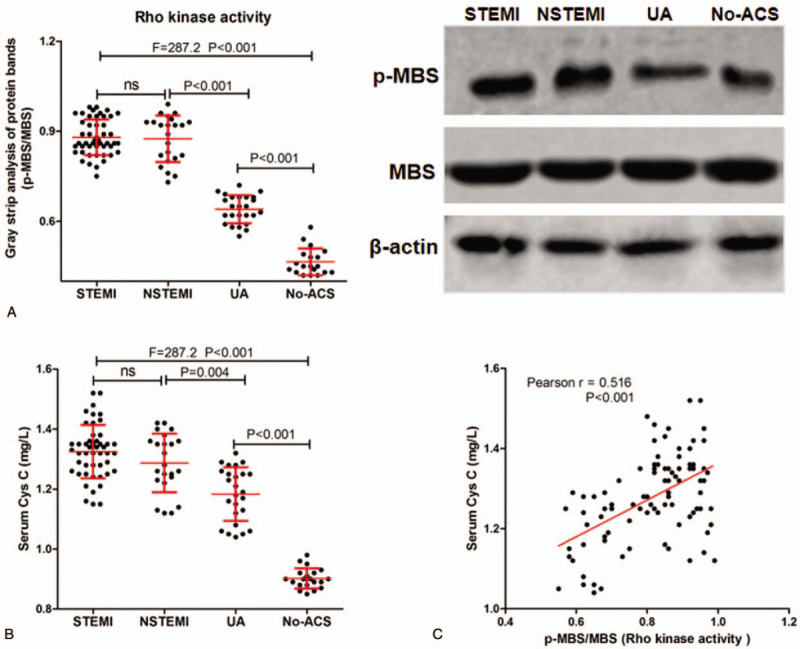
Expression of p-MBS and MBS protein and level of serum Cys C in patients with STEMI, NSTEMI, UA and No-ACS. A-B, Western blot was used to detect the expression of p-MBS / MBS protein in patients with STEMI, NSTEMI, UA and No-ACS (A), and representative protein band (B); C, The level of serum Cys C in patients with STEMI, NSTEMI, UA and No-ACS; D, The correlation between p-MBS / MBS protein and the level of serum Cys C.

The muscle protein binding subunit (MBS) of myosin light chain dephosphorylation enzyme (MLCP) is the major substrate for Rho kinase, and Rho kinase can phosphorylate MBS, so the degree of phosphorylation of MBS can be used to express Rho kinase activity. As showed in Figure 1A, the expression of p-MBS / MBS or the activity of Rho kinase in the leukocyte of STEMI group was the highest, and the No-ACS group was the lowest. Moreover, the activity of Rho kinase in the leukocyte of NSTEMI group was significantly higher than that in UA group, and lower than that in STEMI group, but not significantly.

### 3.2 Level of serum Cys C in patients with or without ACS

The levels of serum Cys C in STEMI, NSTEMI, UA and No-ACS patients were measure, and we found that (Fig. 1B) the level of serum Cys C in STEMI group was the highest, and the No-ACS group was the lowest. And the level of serum Cys C in NSTEMI group was significantly higher than that in UA group, and lower than that in STEMI group, but not significantly. In addition, we also analyzed the correlation between the activity of Rho kinase in the leukocyte of ACS patients and the levels of serum Cys C in ACS patients, and found that (Fig. 1C) there was a positive correlation between leukocyte Rho kinase activity and serum Cys C in ACS patients (r = 0.516, *P* < .001).

### 3.3 Rho kinase activity and serum Cys C were related to inflammation in patients ACS

**Figure 2:**
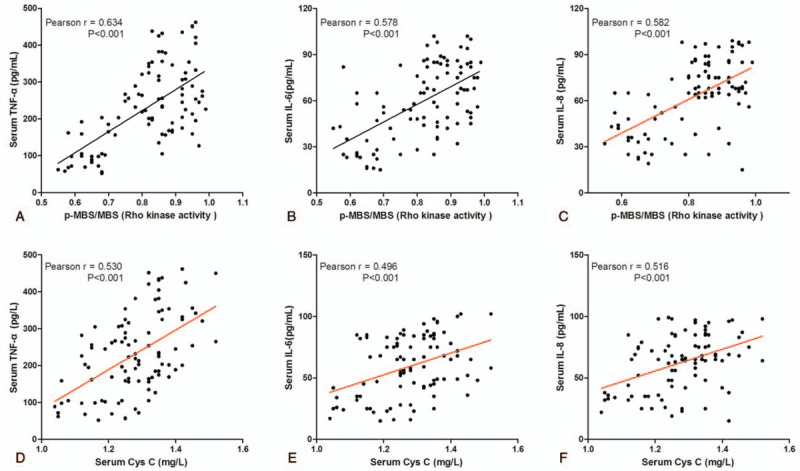
Correlation between Rho kinase activity or serum Cys C and level of Serum inflammatory factor in patients with ACS.

The levels of serum TNF-α, IL-6 and IL-8 were detected for characterization of inflammation in ACS patients, and we analyzed the correlation between the activity of Rho kinase in the leukocyte or the levels of serum Cys C and the levels of serum TNF-α, IL-6, IL-8. And as showed in Figure 2A to C, there was a positive correlation between leukocyte Rho kinase activity and the levels of serum TNF-α (r = 0.634, *P* < .001), IL-6 (r = 0.578, *P* < .001), IL-8 (r = 0.582, *P* < .001) in ACS patients. Furthermore, there was also a positive correlation between the levels of serum Cys C and the levels of serum TNF-α (Fig. 2D, r = 0.634, *P* < .001), IL-6 (Fig. 2E, r = 0.578, *P* < .001), IL-8 (Fig. 2F, r = 0.582, *P* < .001) in ACS patients.

### 3.4 Univariate and multivariate analysis of influencing factors of cardiovascular events in ACS patients

After 12 months of follow-up, 96 patients with ACS were divided into 2 groups based on cardiovascular events, that was with or without cardiovascular event. In this study, the cardiovascular event was 9 deaths, 16 recurrences of ACS and 10 heart failures. As shown in Table 2, age, the proportion of heart failure patients, the level of serum TC and Cys C, the leukocyte Rho kinase activity in ACS patients with cardiovascular events was significantly higher than in ACS patients without cardiovascular events. However, the number of WBC, the proportion of heart failure patients with PCI treatment and statins treatment in ACS patients with cardiovascular events was significantly lower than in ACS patients without cardiovascular events. In addition, univariate and multivariate analysis were used to analyze the influencing factors of cardiovascular events in ACS patients, and the results showed that (Table 3) age, heart failure, the levels of serum TC, PCI treatment, statins treatment, the leukocyte Rho kinase activity (HR = 2.994, 95%CI = 1.328–6.054, *P* < .0001) and the levels of serum Cys C (HR = 1.692, 95%CI = 1.028–2.124, *P* < .0001) were independent influencing factors of cardiovascular events in ACS patients.

**Table 2:**
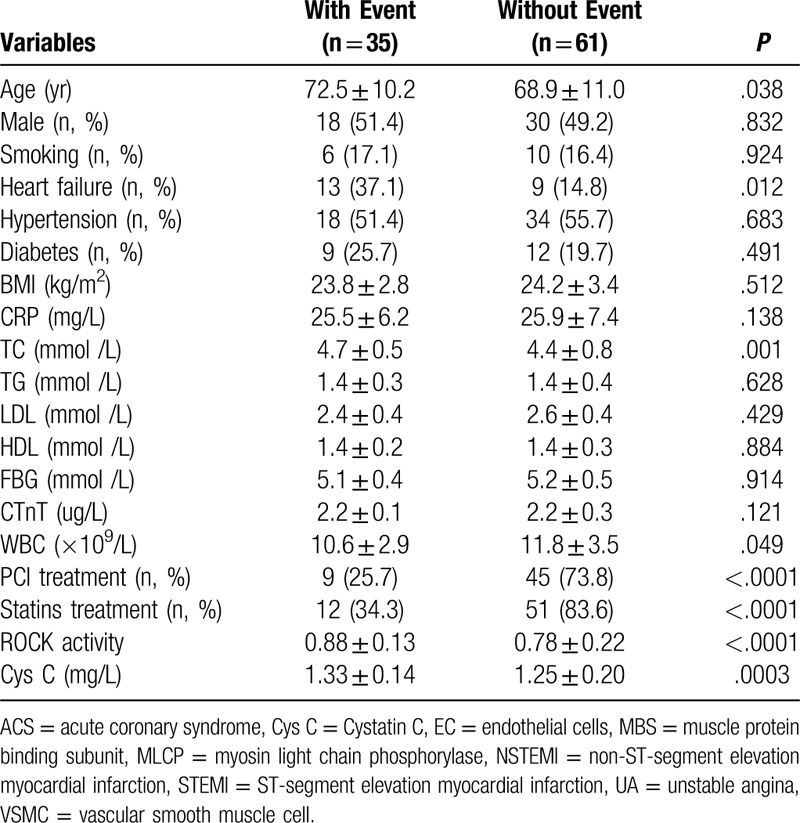
Compare clinical data of ACS patients with or without cardiovascular event.

**Table 3:**
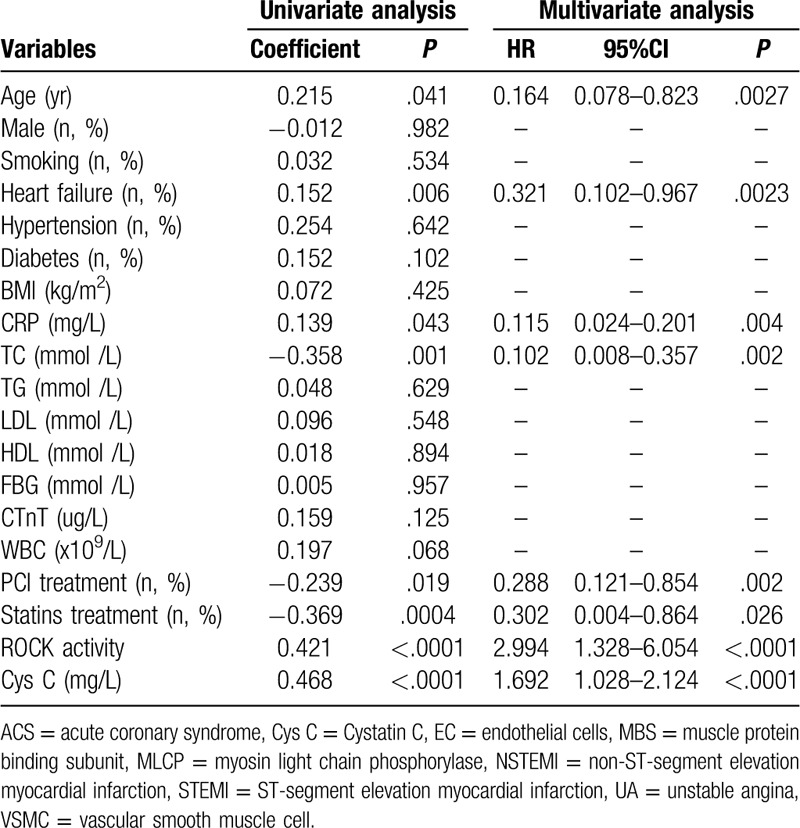
Univariate and multivariate analysis for cardiovascular events of ACS patients.

## 4 Discussion

Rho kinase has been found to be involved in various pathophysiological changes such as morphology, contraction, migration, proliferation, apoptosis, and gene transcription of vascular smooth muscle cells, outer membrane cells, endothelial cells, and cardiomyocytes, and caused a variety of cardiovascular diseases, such as atherosclerosis, hypertension, myocardial ischemia, cardiac hypertrophy and so on.^[10,11]^ Previous studies have found that Rho kinase exerts biological functions by acting on downstream substrate protein molecules, and myosin light chain phosphorylase (MLCP) myosin binding subunit (MBS), ERM protein, adductin, intermediate filament protein and LIM kinase are the substrates of Rho kinase.^[13]^ In this study, we found that the expression of p-MBS / MBS protein was gradually reduced in the STEMI, NSTEMI and UA patients, but all significantly higher than that in No-ASC patients. Because MBS is one of the downstream substrates of Rho kinase and Rho kinase phosphorylates MBS, so the degree of phosphorylation of MBS protein can characterize Rho kinase activity.^[14]^ Therefore, the results of the present study showed that the activity of leukocyte Rho kinase was gradually reduced in the STEMI, NSTEMI, and UA patients, but all significantly higher than that in No-ASC patients.

The proliferation of tunica intima is a major pathological change in vascular restenosis after coronary arteriosclerosis and percutaneous transluminal coronary angioplasty.^[15]^ After vascular injury in ACS patients, a large amount of vasoactive substances are released, which induce the vascular smooth muscle cell (VSMC) to migrate and proliferate to the inner membrane, accompanied by degradation and accumulation of a large number of extracellular matrix complexes, eventually leading to hyperplastic neointimal formation and stenosis of the vascular lumen.^[15]^ And vascular restenosis is one of the main causes of recurrent cardiovascular events in ACS patients. Many studies have found that Rho kinase is involved in the regulation of tunica intima proliferation and restenosis after vascular injury through regulating cell signaling pathway,^[16,17]^ such as Rikitake et al^[16]^ found that Rho kinase regulated the formation of neovascular tunica intima after injury by promoting plasminogen activator inhibitor-1; Funakoshi et al^[17]^ found that Rho kinase can inhibit the migration of monocytes to the tunica intima by directly inhibiting the expression of MCP-1, and finally regulate the intimal repair after vascular injury. As it was recently demonstrated, miR-16 is involved in the activation of the RhoA pathway in endothelial cells (EC) and vascular smooth muscle cells (VSMC) with an adverse impact on progression of atherosclerosis, while its antagonization was able to prevent vascular remodeling.^[18,19]^ Similarly, lots of miRNA were found to play an important role in AMI by affecting EC and VSMC via RhoA pathway.^[20]^ Combined with our results in the present study, Rho kinase activated in ACS patients may affect patient prognosis by affecting EC and VSMC.

In addition, our study also found that the level of serum Cys C had a same trend as the activity of leukocyte Rho kinase in the STEMI, NSTEMI, UA, and No-ASC patients, and there was a positive correlation between leukocyte Rho kinase activity and serum Cys C in ACS patients. Cys C is a group of cysteine protease inhibitors widely found in plants, animals and protozoa, and it not only has anti-viral and protozoal infections, but also promotes cell proliferation.^[14,15]^ And Cys C can act as an inflammatory mediator by activating and chemotactic neutrophils.

Most patients with ACS are caused by unstable plaque rupture and thrombosis, and the inflammatory response plays an important role.^[21]^ And some inflammation-related plasma markers have been shown to be significantly correlated with long-term prognosis in patients with AMI.^[22]^ Previous studies have found that elevated Rho kinase activity can serve as an inflammatory marker for patients with atherosclerosis and peripheral arteriosclerotic disease and found that inhibition of Rho kinase activity inhibited the inflammatory response mediated by the NF-kB pathway.^[23]^ Recently, the role of receptor activator of NF-kB, receptor activator of NF-kB ligand, and the osteoprotegerin system has been recognized as more important in the pathogenesis of cardiovascular disease.^[24]^ In addition, Kataoka et al^[25]^ found the Rho / Rho kinase pathway promoted the formation of atherosclerosis by participating in the regulation of arterial vascular inflammation and NO production in the rat model of atherosclerosis. Cys C regulates inflammatory response in human body is related to the physiological state of the body. In normal physiological state, Cys C regulates vascular inflammation by interfering with the phagocytic and chemotactic functions of granulocytes via inhibiting the activity of endogenous cysteine proteases.^[26]^ In pathological conditions, especially cellular ischemia, when the inflammatory response is initiated, the inflammatory mediators promote the overexpression of cathepsins secreted by vascular smooth muscle cells at arterial elastin lesions, and Cys C regulates vascular inflammation by directly regulating cathepsin activity.^[26]^ In addition, in the present study, undertreatment with statins was an independent predictor of adverse events, and could also explain hyperactivation of inflammation in those patients. It is known that statins have a relevant impact on vascular inflammation and remodeling,^[27]^ and are also able to prevent metabolism-triggered inflammation with an impact on the activation of the Rho pathway through the modulation of protein farnesylation.^[28]^

Patients with diffuse lesions and stenosis of coronary plaques cannot have major cardiovascular events for a long time, but 60% to 70% of patients with ACS develop rapidly from areas of severe stenosis or injury.^[29]^ Therefore, inflammatory cytokines are of great significance in the development, progression, treatment, prognosis, and prediction of severe coronary events.^[29]^ Previous studies have shown that the inflammatory response is closely related to ACS, and the elevated concentrations of inflammatory factors means an increased risk of cardiovascular events and affects prognosis and long-term survival.^[30,31]^ Univariate and multivariate analyses showed that the activity of leukocyte Rho kinase and serum Cys C, which is associated with serum inflammatory factor levels, is an independent risk factor for recurrent cardiovascular events in ACS patients.

## 5 Conclusion

The activity of leukocyte Rho kinase and serum Cys C are increased in patients with ACS, and are related to the development of disease and serum inflammation. Moreover, they are the independent influencing factors of cardiovascular events in ACS patients. However, it should be pointed out that the small sample size is the biggest limitation of this study, which may affect the conclusions of this study.

## 6 Study limitations

This study has some limitations. First, it arose from a single center and therefore was subject to selection bias. Second, the study population was relatively small. Third, lack of long-term observation of neovascular events in patients.

## Author contributions

**Conception/design of study:** Jinhe Zhao, Liang Xu.

**Analysis/interpretation of data:** Li Ma, Wenqin Dai.

**Acquisition of data:** Yongbo Lin, Zhongyuan Zhang, Yunhong Pan, Hongyan Han, Haizhen Jia, Jun Peng.

**Drafting/revising the manuscript:** Li Ma, Wenqin Dai.

**Approval of manuscript:** Jinhe Zhao, Liang Xu.

## Correction

When originally published, reference 24 was missing some information. 2018; 35 (3): 225-232 has been added.
